# A framework of current based defibrillation improves defibrillation efficacy of biphasic truncated exponential waveform in rabbits

**DOI:** 10.1038/s41598-020-80521-9

**Published:** 2021-01-15

**Authors:** Weiming Li, Jingru Li, Liang Wei, Jianjie Wang, Li Peng, Juan Wang, Changlin Yin, Yongqin Li

**Affiliations:** 1 Department of Biomedical Engineering and Imaging Medicine, Army Medical University, 30 Gaotanyan Main Street, Chongqing, 400038 China; 2 Department of Emergency, Southwest Hospital, Army Medical University, Chongqing, 400038 China; 3 Department of Critical Care Medicine, Southwest Hospital, Army Medical University, Chongqing, 400038 China

**Keywords:** Arrhythmias, Ventricular fibrillation

## Abstract

Defibrillation is accomplished by the passage of sufficient current through the heart to terminate ventricular fibrillation (VF). Although current-based defibrillation has been shown to be superior to energy-based defibrillation with monophasic waveforms, defibrillators with biphasic waveforms still use energy as a therapeutic dosage. In the present study, we propose a novel framework of current-based, biphasic defibrillation grounded in transthoracic impedance (TTI) measurements: adjusting the charging voltage to deliver the desired current based on the energy setting and measured pre-shock TTI; and adjusting the pulse duration to deliver the desired energy based on the output current and intra-shock TTI. The defibrillation efficacy of current-based defibrillation was compared with that of energy-based defibrillation in a simulated high impedance rabbit model of VF. Cardiac arrest was induced by pacing the right ventricle for 60 s in 24 New Zealand rabbits (10 males). A defibrillatory shock was applied with one of the two defibrillators after 90 s of VF. The defibrillation thresholds (DFTs) at different pathway impedances were determined utilizing a 5-step up-and-down protocol. The procedure was repeated after an interval of 5 min. A total of 30 fibrillation events and defibrillation attempts were investigated for each animal. The pulse duration was significantly shorter, and the waveform tilt was much lower for the current-based defibrillator. Compared with energy-based defibrillation, the energy, peak voltage, and peak current DFT were markedly lower when the pathway impedance was > 120 Ω, but there were no differences in DFT values when the pathway impedance was between 80 and 120 Ω for current-based defibrillation. Additionally, peak voltage and the peak current DFT were significantly lower for current-based defibrillation when the pathway impedance was < 80 Ω. In sum, a framework of adjusting the charging voltage and shock duration to deliver constant energy for low impedance and constant current for high impedance via pre-shock and intra-shock impedance measurements, greatly improved the defibrillation efficacy of high impedance by lowering the energy DFT.

## Introduction

Ventricular fibrillation (VF) is the most commonly observed presenting rhythm in patients with cardiac arrest^1^. Electrical defibrillation is the method of applying a strong electric field to the fibrillating heart and restoring it to a perfusing rhythm. Thus far, defibrillation by high-energy electric shock is still the only effective technique for terminating VF^2^. Clinical studies indicate that the time interval between patient collapse and shock delivery is the major determinant of outcomes in cardiac arrest patients with VF^3^. After the response time is established, the efficacy of the defibrillation waveform becomes the contributing factor that determines patient outcomes^4^.

The defibrillation waveform is the temporal discharging pattern measured by voltage and current; it interacts with the cardiac electrical activity via its electric field, which is the instantaneous spatial derivative of the shock voltage^5^. The success or failure of a defibrillation depends on the properties of the defibrillation waveform, such as energy, voltage, current, duration, and tilt^6^. Energy has been used to describe the strength of the defibrillation shock for decades in both external and implanted defibrillators, since the amount of energy delivered by a defibrillator is a function of voltage, current, and time^7^. However, there is growing evidence that the success of defibrillation depends not primarily on the delivered energy levels but more on how the energy is delivered^8^. For a defibrillator with the same energy setting, the delivered current (either in terms of peak current or average current) will differ widely among subjects due to the difference in transthoracic impedance (TTI)^9^. TTI, which includes contact impedance and tissue impedance ranged from 33 to 224 ohms, with a mean of approximately 90 to 107 ohms^10–12^. A direct result of the broad variation in TTI is that the output current and energy may be below the threshold required to achieve defibrillation. Clinical studies have confirmed that the defibrillation success rate is negatively correlated with TTI^13^.

Developing techniques for more efficient, low-energy defibrillation is ongoing in today's era of biphasic defibrillation^14–18^. One approach is the waveform shape design. Recent studies suggest that rectilinear biphasic (RLB) and ascending first phase (ASC) waveforms are more efficient than biphasic truncated exponential (BTE) waveforms^15,16^. Another technique entails adjusting the output voltage, current, and energy according to the variance in TTI^17,18^. Current-based defibrillation, which employs current as the therapeutic dosage, has been shown to be superior to energy-based defibrillation in accomplishing shock success when monophasic waveforms are used^19–21^. Contemporary biphasic defibrillators still use energy in joules to describe the strength of a defibrillation shock but utilize impedance compensation techniques to adjust the defibrillation waveform based on patient TTI measurement prior to shock delivery^22^. Although the use of impedance compensation techniques appears to have promising results, a recent clinical study has reported that higher TTI is still associated with greater prevalence of shock failure^23^. Until now, no studies have been conducted to compare defibrillation efficacy between current-based biphasic defibrillation with impedance compensation and energy-based defibrillation. In the present study, we propose a framework of current-based defibrillation for BTE waveforms by gauging pre-shock and intra-shock TTI, and comparing the defibrillation efficacy with that of energy-based defibrillation in a high impedance rabbit model of VF.

## Methods

### Study design and institutional review

This prospective, randomized animal study was designed to compare the efficacy of ventricular defibrillation between current-based and energy-based BTE defibrillation in a rabbit model of VF, in which each animal served as its own control. This model was used because earlier studies demonstrated that it was suitable for assessing fibrillation mechanisms and defibrillation efficacy^24,25^. This study was approved by the Research Council and Animal Care and Use Committee of the Army Medical University. All animals received humane care in compliance with the Principles of Laboratory Animal Care and the Guide for the Care and Use of Laboratory Animals.

### Framework of the proposed current-based defibrillation

**Figure 1:**
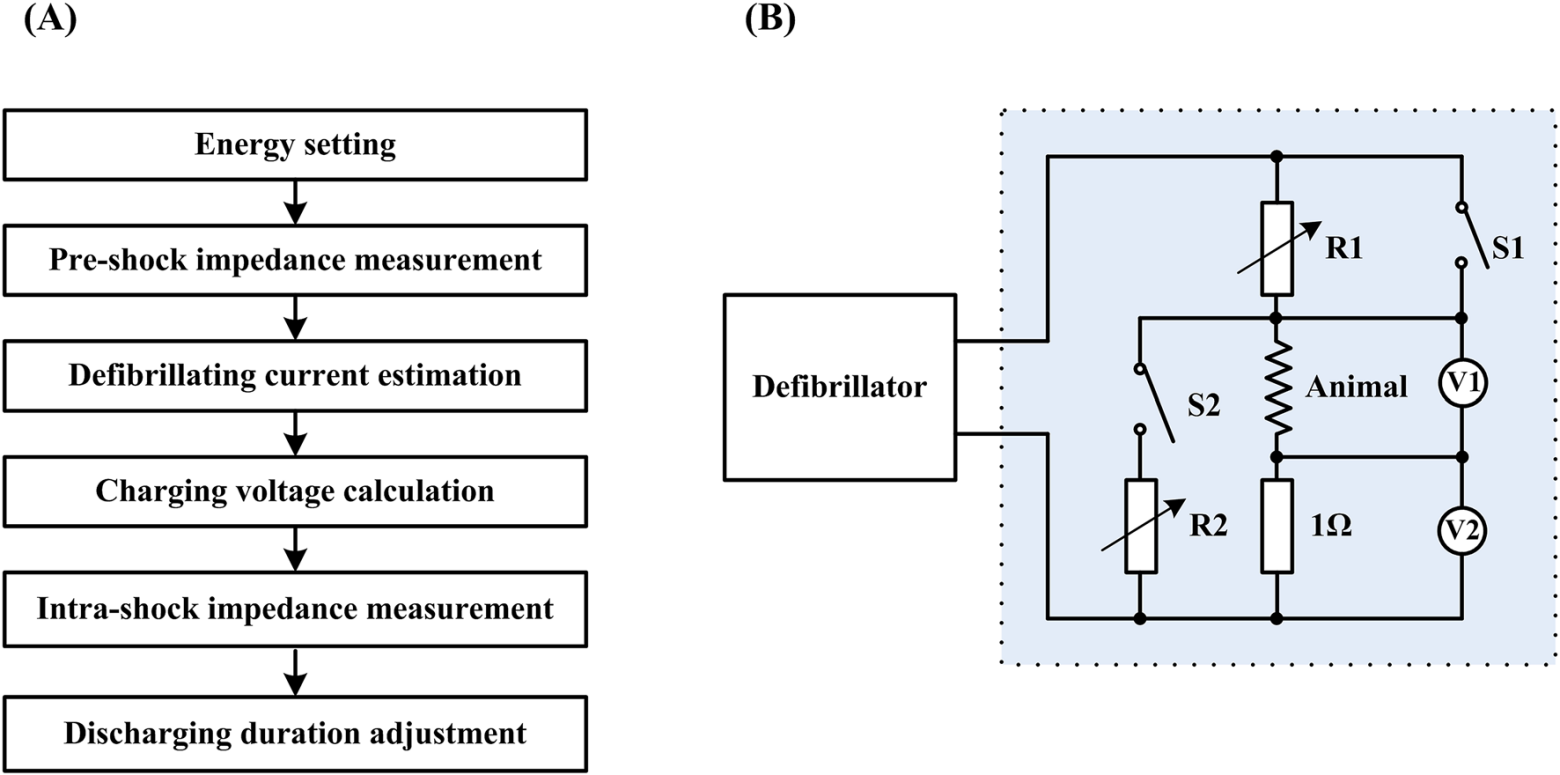
(**A**) Implementation of current-based defibrillation; (**B**) setup for different pathway impedance simulation.

The principle of the proposed current-based defibrillation is composed of two steps: (1) measuring TTI prior to administering the charge and adjusting the charging voltage to deliver the desired current; and (2) measuring TTI during the initial discharging period and adjusting the pulse duration to deliver the desired energy. A flowchart of the defibrillation waveform parameter setup is shown in Fig. 1A. First, the defibrillator is started with a user-defined (or default) energy setting, the same as traditional energy-based biphasic defibrillators. Second, the pre-shock TTI (*R*_*AC*_) is gauged by the delivery of a 1 mA 30-kHz alternating current between the two pads when the charge button is pressed. The target average current *I*_*a*_ is computed based on the measured pre-shock TTI using the following formula:

$$I_{a} = \left\{ {\begin{array}{*{20}c} {\sqrt {{E \mathord{\left/ {\vphantom {E {R_{AC} t_{0} }}} \right. \kern-\nulldelimiterspace} {R_{AC} t_{0} }}} ,} & {\quad {\text{if}}\;\;R_{AC} \le R_{T} } \\ {\sqrt {{E \mathord{\left/ {\vphantom {E {R_{T} t_{0} }}} \right. \kern-\nulldelimiterspace} {R_{T} t_{0} }}} ,} & {\quad {\text{if}}\;\;R_{AC} > R_{T} } \\ \end{array} } \right.$$

where *E* is the energy setting, *R*_*T*_ is the threshold of high impedance, *R*_*AC*_ is the measured pre-shock TTI and *t*_*0*_ is the default shock duration. Third, based on the relationship between the final current *I*_*f*_ and peak current *I*_*p*_ of the BTE waveform:

$$I_{f} = I_{p} \times e^{{{{ - t_{0} } \mathord{\left/ {\vphantom {{ - t_{0} } {RC}}} \right. \kern-\nulldelimiterspace} {RC}}}}$$

the target average current *I*_*a*_ can be calculated as

$$I_{a} = (I_{p} + I_{f} )/2$$

based on the fact that current decays linearly with time when the discharge time is less than the time constant of the RC charge/discharge circuit. Hence, the peak current is

$$I_{p} = {{2 \times I_{a} } \mathord{\left/ {\vphantom {{2 \times I_{a} } {(1 + e^{{ - t_{0} /R_{AC} C}} )}}} \right. \kern-\nulldelimiterspace} {(1 + e^{{ - t_{0} /R_{AC} C}} )}}$$

where *C* is the capacitance value of the defibrillator. Fourth, the charging voltage is determined based on the target peak current and pre-shock TTI:

$$V_{p} = I_{p} R_{AC}$$

Finally, intra-shock TTI (*R*_*DC*_) is established during the initial period of the shock, based on the delivered voltage and current. The total shock duration will be adjusted using the measured intra-shock TTI to maintain the desired output energy:

$$E_{d} = R_{DC} \int_{0}^{{t_{d} }} {i^{2} (t)} dt$$

where *E*_*d*_ is the delivered energy, *R*_*DC*_ is the intra-shock TTI, and *t*_*d*_ is the delivered shock duration.

### Defibrillation system and variable pathway impedance simulation

Current-based defibrillation was delivered with a custom designed research prototype, delivering a low tilt BTE waveform utilizing a capacitor of 210 μF. A larger capacitor was employed because it reduced the peak current and delivered a more constant current^26^. In the current study, *C* was 210 μF, *R*_*T*_ was 100 ohms and *t*_*0*_ was 8 ms. The ratio between the phase 2 duration and the phase 1 duration is set to 2/3, since previous studies have demonstrated that the optimal phase-duration ratio is between 30 and 70% when a larger capacitor is used^27,28^.

Energy-based defibrillation was delivered with a HeartStart XL + defibrillator (Philips Healthcare, Seattle, WA, USA). The defibrillator produced a BTE waveform and compensated for the increased TTI by prolonging the duration of the defibrillation shock. The duration increased from 5.8 to 20.8 ms based on the measured TTI^29^.

The TTI of the rabbits used for the experiment was approximately 80–120 ohms, which covers only a small range of that of adult humans. To simulate a wide-ranging variable impedance rabbit model, a pathway impedance network consisting of two switches, two adjustable resistors, a precision 1-Ω current sensor resistor, and the animal was connected to the output of the defibrillators. As shown in Fig. 1B, R1 represents the serial electrode-tissue interface resistance while R2 embodies the parallel resistive components of the thorax. The pathway impedance will be high if S1 and S2 are off and will be low if S1 and S2 are on. The pathway impedance can also be finely tuned by adjusting R1 and/or R2. This setup configuration can control a shock's success or failure by simulating the defibrillation of low (< 80 Ω), medium (80–120 Ω) and high (> 120 Ω) impedance patients using a predefined defibrillator dosage.

**Figure 2:**
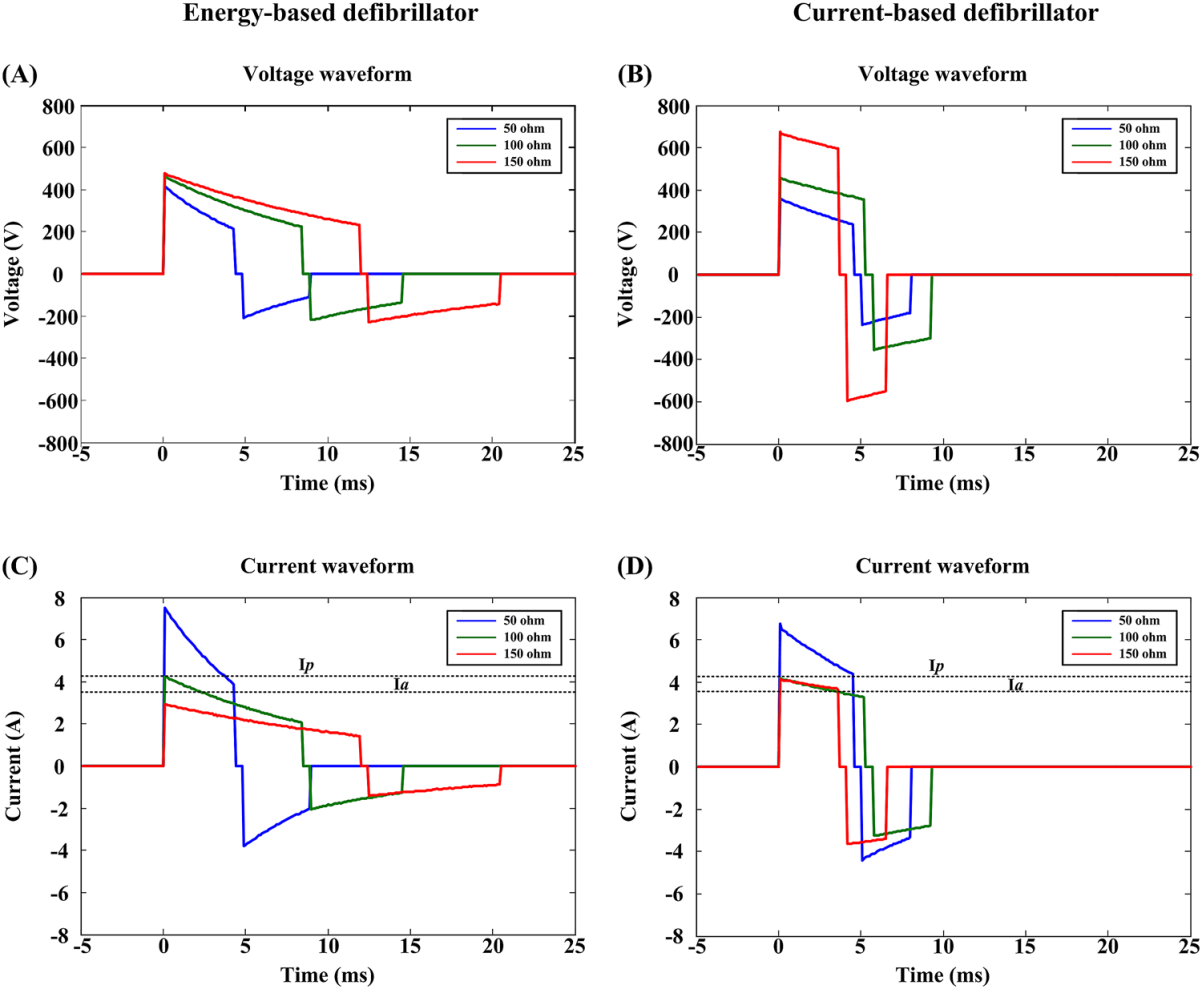
Defibrillation voltage (**A**,**B**) and current (**C**,**D**) waveforms of current-based and energy-based defibrillations for a 10 J test shock when the pathway impedance was 50, 100 and 150 ohms respectively. I_a_ and I_p_ indicate the target average and peak currents described in Eqs. (1) and (4).

**Figure 3:**
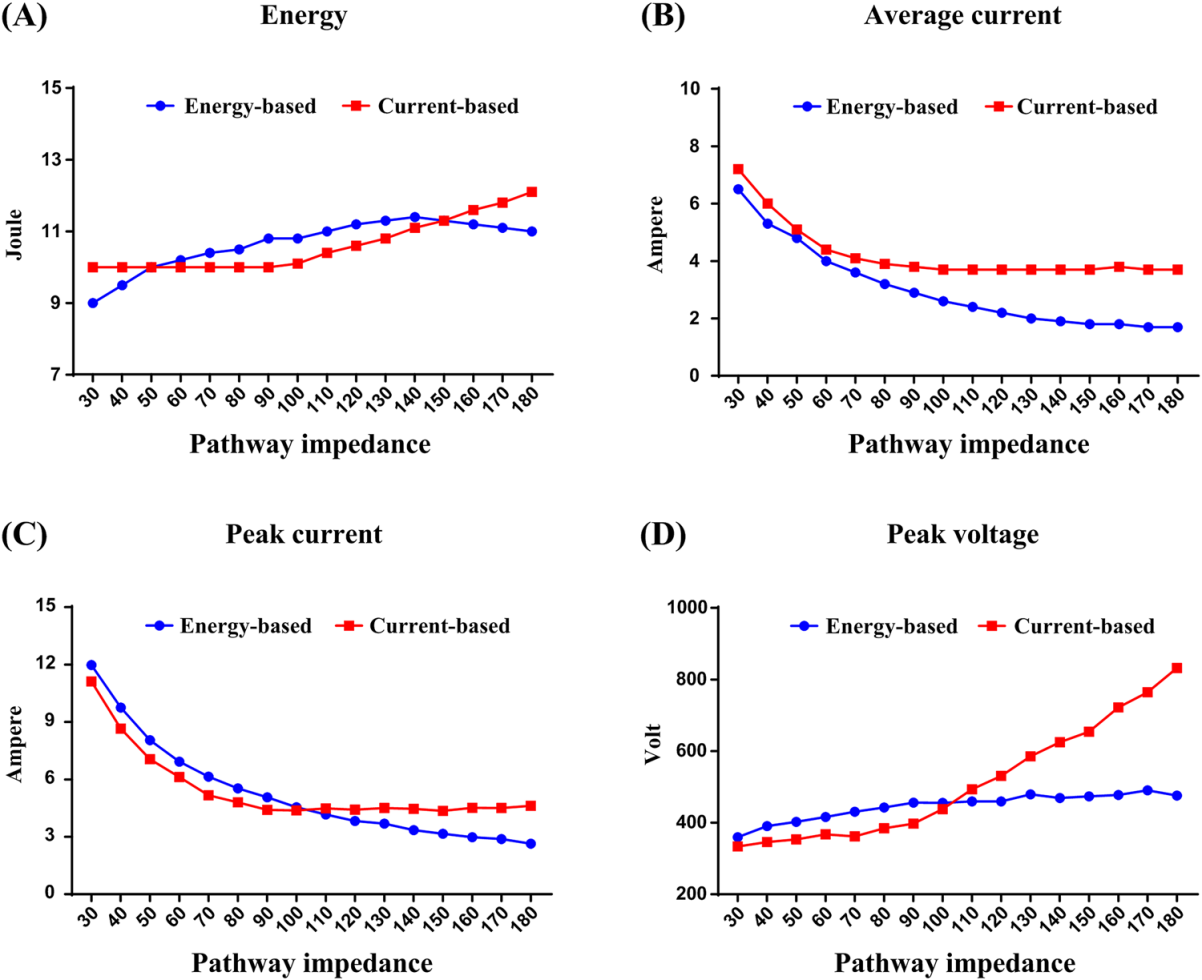
The output energy (**A**), average current (**B**), peak current (**C**), and peak voltage (**D**) value for a 10 J test shock in relationship to the pathway impedance in current-based and energy-based defibrillation.

Figure 2 illustrates the defibrillation waveforms delivered through the two defibrillators when the pathway impedance was 50, 100, and 150 Ω at an energy setting of 10 J. Figure 3 portrays the defibrillation parameters including energy, average current, peak current and peak voltage value in relationship to the pathway impedance of the two defibrillators. Unlike energy-based defibrillators, the current delivered by the current-based defibrillator did not decay with the increased pathway impedance when it was greater than 100 ohms. At the same time, both energy and peak voltage increased linearly with the increments of the pathway impedance for the current-based defibrillator.

### Animal preparation

Twenty-four New Zealand rabbits of both sexes (10 males), weighing between 1.8 and 2.4 kg, were utilized for this study. The animal preparation procedure was similar to that of our previous study^25^. The animals were anaesthetized via ear vein injection of sodium pentobarbital at a dose of 30 mg/kg and placed on a surgical board in the supine position. After intubation with an 11-gauge cannula, the animals were mechanically ventilated with room air at a tidal volume of 0.60 mL/100 g (ALC-V8, Alcott Biotech Co. Ltd, Shanghai, China). A PE-160 catheter was inserted into the surgically exposed right femoral artery to measure arterial pressure. A 5F bipolar pacing catheter (St. Jude Medical, Minnetonka MN, USA) was advanced from the right external jugular vein into the right ventricle to induce VF. Three needle electrodes were applied to the right upper, left upper, and lower limbs to record the electrocardiogram (ECG). A pair of external defibrillation pads with a diameter of 40 mm was fixed on the chest, with the positive pad placed on the right side and the negative pad placed on the left side, so that the heart was directly interposed between the two pads. An electrically conductive adhesive gel (SIGNA GEL, Parker Laboratories Inc, Fairfield, NJ, USA) was interposed between the pads and the skin to ensure close contact.

### Experiment procedures

The experimental procedures were established in our prior research^25,30^. Briefly, VF was induced by delivering a 50-Hz AC current of 3–5 mA into the right ventricular endocardium and was confirmed by the sudden drop in arterial pressure and the absence of a pulse. Stimulation was applied for 60 s in order to prevent spontaneous defibrillation. After 90 s of untreated VF, a test shock was delivered from a randomly selected defibrillator. Defibrillation success was defined as the return of a perfusing rhythm within 5 s after attempted defibrillation. If VF was not terminated, a 3 J rescue shock, delivered from the other defibrillator, was applied directly to the animal. A waiting period of a minimum of 5 min was required between each test shock to insure haemodynamic stability. A 5-step up-and-down protocol was used to determine the defibrillation threshold (DFT) for each defibrillator system^30^. The procedure was repeated 3 times to determine the DFTs with low, medium and high pathway impedance. At the end of the experiment, the animals were euthanized by a lethal intraperitoneal injection of sodium pentobarbital (150 mg/kg).

### Randomization and blinding

A total of 30 fibrillation and defibrillation events were investigated for each animal. The 30 test shocks included 15 shocks from each defibrillator system. The 15 test shocks were 5 shocks at 3 different pathway impedance levels. To control for possible experimental bias caused by repetitive VF induction and defibrillation, the primary investigator randomly chose one pathway impedance level from a sealed envelope then selected the defibrillator used for testing from another sealed envelope. The energy setting for the first step was set at 3 J and R1/R2 was set to maintain the pathway impedance network at a low, medium or high level. The energy setting, together with the R1 and/or R2 of subsequent shocks, was adjusted to decrease or increase the delivered energy to the animals by nearly 25%, depending on the success or failure of the preceding step. The co-investigator who induced VF and recorded the experimental measurements and outcomes was blinded to the defibrillator selection and pathway impedance settings. The results for the shock parameters and the defibrillation outcomes were incorporated into a final dataset for analysis after the experiment was finished.

### Measurements

Arterial pressure, heart rate, and body temperature were continuously measured, as described in our previous study^25^. Arterial pressure and conventional lead II ECG were recorded using a multiparameter monitor (Model 90369, Spacelabs, Snoqualmie, WA, USA). Rectal temperature was monitored with a thermocouple probe (TH-212, Bjhocy science and technology Co. Ltd., Beijing, China) that was placed into the rectum and maintained between 37.0 and 37.5 °C with a heating lamp. The defibrillation voltage and current waveforms delivered to the animal (V1 and V2 in Fig. 1B) were simultaneously recorded during each attempted shock with a high speed USB data acquisition system (DI-730-USB, Dataq instruments, Akron, OH, USA) at a sample rate of 10 kHz. To evaluate defibrillator safety, the heart rate and mean arterial pressure (MAP) before and after each fibrillation/defibrillation event were compared.

### Statistical analysis

Data were reported as the mean ± standard deviation. The DFTs of energy, peak voltage, peak current, and average current served as the primary measurement outcomes for defibrillation efficacy^25^. For measurements within groups, a two-sided, paired Student's *t*-test was employed. The association between pre-shock TTI and intra-shock TTI was gauged using Pearson's correlation. A *p* value < 0.05 was considered significant.

## Results

A total of 720 testing shocks were delivered, and 52.9% of the attempts (381 test shocks, 181 from energy-based defibrillation and 200 from current-based based defibrillation) were successful. Heart rate (230.5 ± 42.1 beats/min vs. 243.5 ± 34.1 beats/min, *p* = 0.234) and MAP (72.3 ± 13.9 mmHg vs. 69.7 ± 10.6 mmHg, *p* = 0.406) did not show significant differences between the beginning and end of the experiment. The determined pre-shock TTI was relatively lower than the intra-shock TTI for current-based defibrillation (99.2 ± 9.9 Ω vs. 103.1 ± 13.1 Ω, *p* < 0.001), and the correlation coefficient between the pre-shock TTI and the intra-shock TTI was 0.74 (*p* < 0.001). When all of the test shocks were considered, the average current DFT (0.99 ± 0.43 A vs. 0.99 ± 0.46 A, *p* = 0.977) was equivalent between the two defibrillators, but the energy (0.72 ± 0.53 J vs. 1.04 ± 1.03 J, *p* = 0.005), peak current (1.09 ± 0.46 A vs. 1.32 ± 0.60 A, *p* < 0.001), and peak voltage (112.7 ± 40.1 V vs. 130.1 ± 46.5 V, *p* < 0.001) DFT were significantly lower for current-based defibrillation.

**Figure 4:**
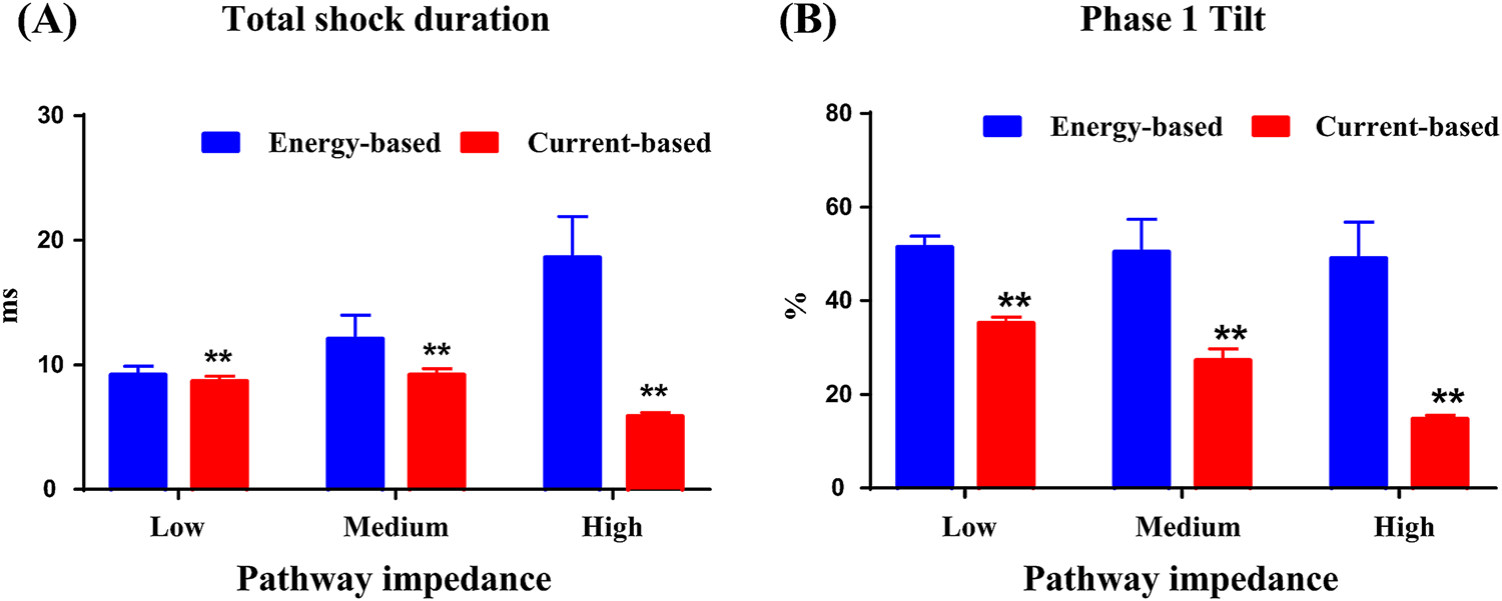
Total shock duration (**A**) and the phase 1 tilt (**B**) of the current-based and energy-based defibrillations at different pathway impedances.

The total impedance was 61.9 ± 2.1 Ω, 96.6 ± 7.0 Ω, and 163.5 ± 10.1 Ω for the low, medium and high pathway impedance settings respectively. Resistor R1 was 23.7 ± 10.7 Ω, 45.5 ± 14.9 Ω and 103.1 ± 15.4 Ω. Resistor R2 was 72.8 ± 43.2 Ω, 90.2 ± 38.5 Ω, and 121.8 ± 48.6 Ω. These configurations result in the low, medium and high pathway impedances respectively. As shown in Fig. 4, the waveform delivered by the current-based defibrillator had a significantly shorter total shock duration and lower waveform tilt than that delivered by the energy-based defibrillator. The haemodynamic data measurements at each fibrillation/defibrillation event for different pathway impedances are listed in Table 1. There were no significant differences in animal TTI, MAP, or HR between the two defibrillators.

**Table 1:** Haemodynamic data measurements at each fibrillation/defibrillation event for different pathway impedances.

Group/defibrillator	TTI (Ω)	MAP (mmHg)		HR (beats/min)	
		Before	After	Before	After
**Low pathway impedance (< 80** **Ω)**					
Energy-based	98.7 ± 9.4	68.8 ± 9.0	69.2 ± 8.8	243.3 ± 32.0	244.4 ± 32.8
Current-based	101.2 ± 9.5	69.4 ± 9.6	69.2 ± 9.7	242.2 ± 32.6	240.9 ± 32.2
**Medium pathway impedance (80–120** **Ω)**					
Energy-based	102.7 ± 12.7	69.9 ± 9.1	69.4 ± 9.0	247.7 ± 29.9	251.9 ± 34.1
Current-based	104.2 ± 12.3	69.6 ± 8.7	69.7 ± 8.9	248.4 ± 32.4	248.6 ± 33.2
**High pathway impedance (> 120** **Ω)**					
Energy-based	99.7 ± 10.1	70.0 ± 7.8	69.5 ± 7.8	246.6 ± 28.6	246.2 ± 27.3
Current-based	100.5 ± 9.1	69.1 ± 7.8	68.3 ± 8.5	248.7 ± 31.5	246.8 ± 27.7

**Figure 5:**
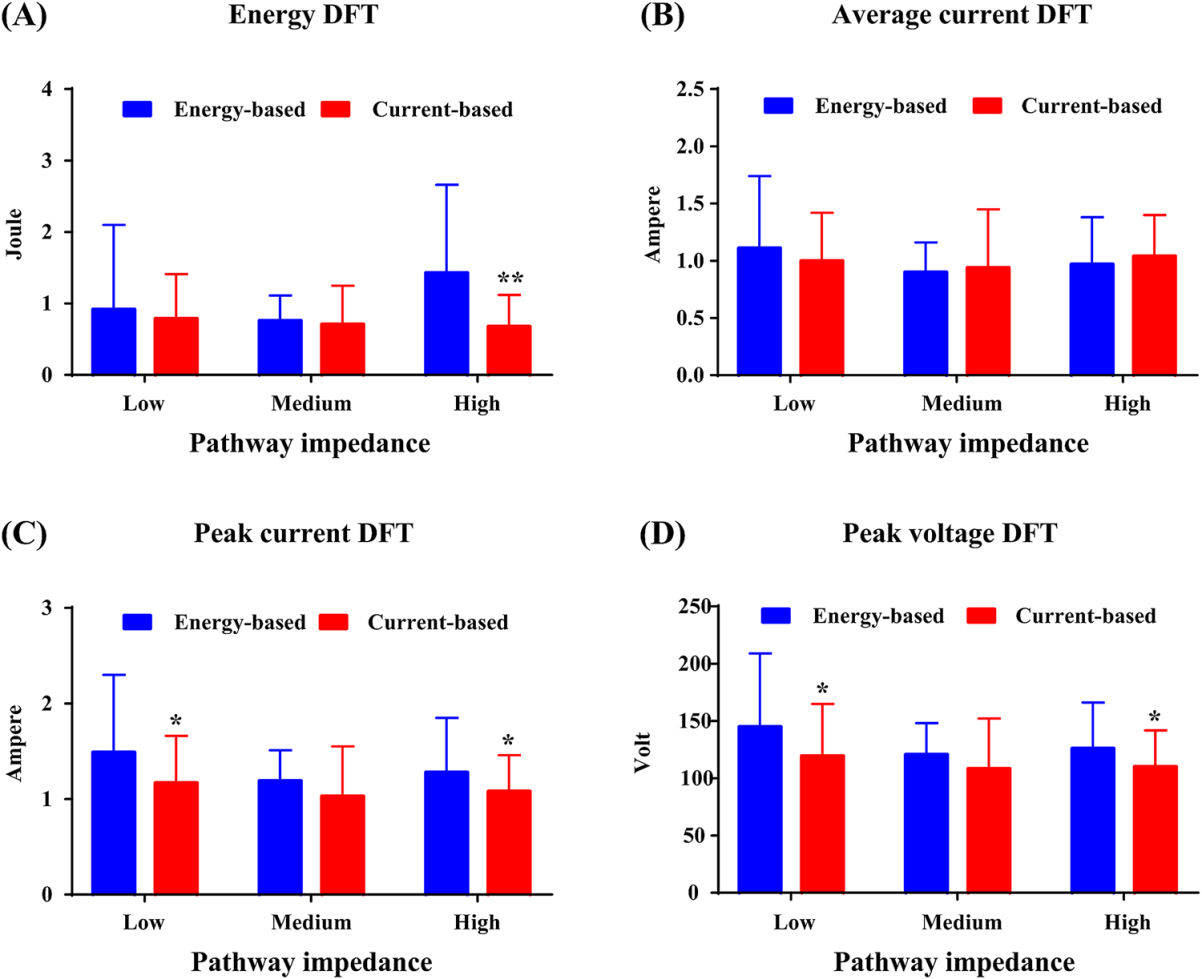
Energy defibrillation threshold (DFT) (**A**), the average current DFT (**B**), the peak current DFT (**C**) and the peak voltage DFT (**D**) for current-based and energy-based defibrillation at different pathway impedances.

The DFT values at different pathway impedances are summarized in Fig. 5. For energy-based defibrillation, the average current DFT did not differ significantly at different pathway impedances, but the peak current DFT was considerably higher for low pathway impedance than for medium pathway impedance (1.49 ± 0.81 A vs. 1.19 ± 0.32 A, *p* = 0.039). The mean within-animal DFT standard deviation was 0.25 ± 0.24 A for the average current and 0.48 ± 0.23 A for the peak current. For current-based defibrillation, neither the average current DFT nor the peak current DFT differed significantly at different pathway impedances. The mean within-animal DFT standard deviation was 0.24 ± 0.21 A and 0.25 ± 0.22 A for the average and peak currents, respectively. Compared with energy-based defibrillation, the energy (0.68 ± 0.44 J vs. 1.43 ± 1.23 J, *p* = 0.001), peak current (1.08 ± 0.38 A vs. 1.28 ± 0.57 A, *p* = 0.032), and peak voltage (110.2 ± 31.6 V vs. 126.0 ± 40.1 V, *p* = 0.042) DFT were markedly lower for current-based defibrillation when the pathway impedance was high. However, there were no differences in DFT values between the two defibrillators when the pathway impedance was medium. Additionally, the peak current (1.17 ± 0.49 A vs. 1.49 ± 0.81 A, *p* = 0.024) and peak voltage (119.6 ± 45.2 V vs. 145.0 ± 63.9 V, *p* = 0.017) DFT were significantly lower for current-based defibrillation in low pathway impedance.

## Discussion

The present study compared the defibrillation efficacy between current-based and energy-based defibrillation in terms of DFT over a wide-range of pathway impedances in a rabbit model of VF. We found that the average current DFT did not differ between the two defibrillators, but the energy, peak current, and peak voltage DFTs were significantly reduced for current-based defibrillation when the pathway impedance was high.

Successful defibrillation is achieved by delivering a high-energy shock comprised of the brief pulse of an electrical field, with adequate current and a phasic duration capable of terminating VF. Although shock efficacy is steadily improving and defibrillation has become the only effective therapy against VF, severe side effects (e.g. electroporation, contractile and electrical dysfunction, etc.) may accompany the treatment of VF^31,32^. Therefore, developing more efficient defibrillators that can reduce the energy required to terminate VF while limiting the risk of high-energy shock induced cardiac injury remains a subject of extensive research.

As it is accepted that current is a better indicator of shock success and therapeutic dose selection than energy, the concept of current-based defibrillation has been proposed, and the techniques of current-based defibrillation have been investigated in monophasic defibrillations. In an animal study, Geddes et al*.* demonstrated that it is feasible to predict TTI prior to a defibrillation shock by delivering a low-current, high-frequency, sinusoidal excitation current to the electrodes^33^. Using this technique, Kerber et al*.* validated that the predicted TTI was highly correlated with the actual impedance of a damped sinusoidal waveform in 19 patients who received 66 defibrillation shocks and the mean TTI was 78.1 Ω^34^. Based on the observation that the current DFT was independent of TTI in canines, Lerman et al*.* suggested redefining DFT in terms of peak current (rather than energy)^35^. They also compared the efficacy of current-based and energy-based defibrillation in 86 patients who developed VF during programmed electrical stimulation. The clinical results indicated that both energy and current DFTs were remarkably reduced for current-based defibrillation at equivalent shock success rates^19^. Machin et al*.* designed a damped sinusoidal wave defibrillator capable of delivering a constant peak discharge current over a wide range of patient impedances^36^. The maximal current available was 45 A, the maximal charge voltage was 9 kV, and the maximal stored energy was 745 J. However, Kerber et al*.* found that the success rate for patients with high TTI did not significantly improve when compared with energy-based defibrillation in a prospective multicenter clinical trial^37^. There are three possible reasons for the unimproved defibrillation efficacy: (1) the variance between the predicted and actual TTI was large since the TTI was inversely related to the defibrillation voltage, especially for the ultra high charging voltage applied to monophasic waveforms^36^; (2) both pre-shock and intra-shock TTI were affected by the electrode position and force for the hand-held, circular, stainless paddle electrodes^19^; (3) the relationship between current and energy threshold/dosage for the damped sinusoidal waveform was undetermined^6^.

Unlike monophasic waveforms, biphasic waveforms deliver voltage/current in two directions, with the first phase charging the cell membranes and the second phase removing residual charges^37^. Clinical trials proved that biphasic waveforms have greater shock efficacy and fewer side effects for ventricular defibrillation^38^. Unfortunately, the TTI for low-energy, biphasic defibrillation shocks is significantly higher than that measured in high-energy monophasic shocks^11,12^. Instead of using the concept of current-based defibrillation, biphasic defibrillators still use energy as the therapeutic dosage. In order to compensate for variation in TTI, biphasic defibrillators were implemented with the impedance compensation technique, whereby the shock amplitude and/or duration was adjusted according to the measured TTI^9^. A significant improvement in the defibrillation success rate was observed when the impedance compensation technique was applied for patients with high TTI^11,39^. However, the waveform design and the impedance compensation method may differ markedly among manufacturers. Using the high impedance porcine model of VF, we demonstrated that current-based compensation, by maintaining a current with a fixed shock duration, is more effective than duration-based compensation by prolonging shock duration when the TTI is greater than the average^9^. The experimental outcomes support the design of current-based biphasic defibrillators as an alternative approach to energy-based ones in order to avoid an inappropriate defibrillation dose when the TTI varies among patients^40,41^.

In the current study, we developed a framework of current-based, biphasic defibrillation that not only ensures the minimum energy required for defibrillation but also guarantees the minimum current required for defibrillation. On the one hand, the designed defibrillator delivered a constant current for high impedance but delivered a constant energy for low impedance, which is different from the current-based, monophasic defibrillator, which delivers a predefined current for all patients. On the other hand, the designed defibrillator adjusted the output by increasing the shock voltage but shortening the shock duration, which is different with the impedance compensation method of prolonging shock duration. The experimental findings show that the proposed method is superior to energy-based, biphasic defibrillation in terms of DFT. The enhanced defibrillation efficacy can be explained using the following factors.

First, current is the main parameter determining whether ventricular defibrillation is successful, whereas energy is the main parameter determining the shock-induced dysfunctions experience by the patient. Although the shock amplitude, shock strength, and waveform tilt differed between the two investigated defibrillators, there were no differences in the average current DFT at different pathway impedances. This is consistent with our previous findings that the average current is the major determinant of defibrillation efficacy^41^. The designed defibrillator thus required less peak current and peak voltage to output the same level of average current due to its low tilt.

Second, shock duration is another important determinant of defibrillation efficacy for biphasic waveforms^42^. An earlier study reported that an optimal biphasic waveform with the first phase duration lasting between 4 and 10 ms, and the second phase duration being shorter than the first one resulted in the lowest energy DFT^43^. In addition to the decreased current, another possible reason for the decreased defibrillation success rate for energy-based defibrillation in higher TTI patients is that the shock duration lasts beyond the optimal duration^23^. Since energy is the product of the square of average current and total shock duration, the energy requirements for waveforms with shorter phase durations will be significantly lower than those for waveforms with prolonged durations. The increased average current, together with decreased shock duration, led to a significantly lower DFT for current-based defibrillation when the pathway impedance was high.

Although the concept of current-based defibrillation is appealing, and the transition to current-based defibrillation was encouraged by the 2010 American Heart Association guidelines for CPR and emergency cardiovascular care science, switching from traditional energy-based defibrillation to current-based defibrillation is not easy^44^. Because the therapeutic dosage and defibrillation success are not linearly related, there is no distinct cut-off value for current or energy. Additionally, a drawback of using current as a therapeutic dosage is that the delivered energy will be below the DFT when the TTI is low. Given that the clinicians care about the defibrillation outcomes more than the defibrillator's technical aspects, we still used energy as the therapeutic dosage when impedance was low. However, if the impedance becomes greater than average, the defibrillator will automatically change to the current mode without the operator's intervention. This solution can be accepted by both manufacturers and clinicians for the convenience of manufacturing defibrillators and making them easy to use.

There are several limitations to our study. First, the 90-s duration of VF was much shorter than the long-term VF that occurred in victims of cardiac arrest. Long-term VF may have different maintenance and termination mechanisms than short-term VF. Second, electricity-induced VF model differs from the ischaemic-induced VF; the simulated high pathway impedance was also different from the high impedance of patients observed in clinical situations. Additionally, the physiological structure of the rabbit is quite different from that of the human body. The experimental results therefore need to be further verified in large animal models that are closer to the human body structure. Third, defibrillation efficacy was compared between BTE waveforms, and was not validated for other biphasic waveforms such as the RLB and ASC waveforms. Hence, whether the framework of the design can be applied to these biphasic waveforms is undetermined. Fourth, a larger capacitor was used to deliver the low tilt waveform with the high average current and the low peak current. This design is more advantageous at higher impedances for external defibrillation, but might not be suitable for implantable defibrillators where the impedance is lower and energy is more important for defibrillation. For patients with low impedance, the DFT may increase due to the increased time constant of the defibrillation system.

## Conclusions

The present study proposed a framework of current-based, biphasic defibrillation by adjusting the charging voltage and shock duration, delivering constant energy for low impedance and constant current for high impedance. Current-based defibrillation outperforms impedance-compensating, energy-based defibrillation for BTE waveforms by lowering the energy DFT in a high impedance VF rabbit model.
